# Gastrocnemius muscle flap with vancomycin/gentamicin-calcium sulfate and autogenous iliac bone graft for the phase I treatment of localized osteomyelitis after tibial plateau fracture surgery

**DOI:** 10.1186/s13018-021-02496-1

**Published:** 2021-05-27

**Authors:** Weiwei Ruan, Menglu Li, Qiaofeng Guo, Bingyuan Lin

**Affiliations:** 1 Department of Orthopedics, Tongde Hospital of Zhejiang Provincial, Zhejiang Academy of Traditional Chinese Medicine, No. 234 Gucui Road, Hangzhou 310002 Zhejiang, People’s Republic of China; 2 Institute of Cancer Research and Basic Medical Sciences, Cancer Hospital of University Academy of Sciences, Zhejiang Cancer Hospital, Hangzhou, People’s Republic of China

**Keywords:** Osteomyelitis, Bone graft, Phase I, Gastrocnemius muscle flaps, Tibial plateau fractures, Vancomycin

## Abstract

**Purpose:**

To investigate the clinical effect of gastrocnemius muscle flaps combined with vancomycin/gentamicin-calcium sulfate combined and autologous iliac bone graft in the phase I treatment of traumatic focal osteomyelitis (Cierny-Mader type III) after tibial plateau fracture surgery.

**Methods:**

From July 2009 to January 2018, 35 patients with localized osteomyelitis (Cierny-Mader type III) who met the inclusion criteria were followed up and treated. All patients were infected after undergoing internal fracture fixation surgery. Among them, 18 cases were plate-exposed, 14 cases were due to sinus tracts, two were due to skin necrosis, and one was bone-exposed. We treated patients with several measures. All cases were then followed up. The follow-up indicators included Hospital for Special Surgery knee scores (HSS), the time of laying drainage pipe, bone healing time, infection control rate, and the incidence of nonunion and other complications.

**Results:**

All patients were followed up for 24–60 months. None of them underwent amputation. For repairing soft tissue defects, 17 cases were covered with a muscle flap using the medial head of gastrocnemius alone, 15 cases were treated with the lateral head of gastrocnemius muscle, and three cases were covered with the combination of the two heads. Compared to the preoperative score, we found that the average HSS improved at the 1-year and 2-year follow-up (54 vs. 86 vs. 87).

**Conclusion:**

Using a gastrocnemius muscle flap combined with vancomycin/gentamicin-calcium sulfate and autogenous iliac bone was an effective method for the phase I treatment of osteomyelitis (Cierny-Mader type III) after tibial plateau fracture surgery. In the primary treatment of focal traumatic osteomyelitis, it can decrease the treatment time, number of surgeries, pain of patients, time of bone healing, postoperative exudation, and infection recurrence rate and increase the healing bone’s strength.

## Introduction

Tibial plateau fractures are intra-articular fractures that are mostly secondary to high-energy injuries. The basic principles of treatment include anatomical reduction, firm fixation, and early functional training [1]. Therefore, surgical treatment using internal fixation is often the choice of treatment. However, due to the serious injuries on the soft tissues around the fracture, improper selection of operation method and timing, and improper operation technique, this surgical technique becomes complicated due to a series of related complications, such as poor wound healing, infections (such as infectious osteomyelitis), soft tissue defects, exposure of implants, and bone nonunion [2]. Because the soft tissue in front of the tibia is weak, tibia is one of the most susceptible sites of chronic infectious diseases in long bones of extremities [3, 4]. Since the infection recurrence rate as well as the amputation risk are high, the clinical efficacy of osteomyelitis after tibial plateau fracture is still unsatisfactory [5, 6]. The traditional treatment of chronic osteomyelitis is a staged treatment regimen represented by antibiotic bead chain implantation, with a two-stage autologous cancellous bone graft [7–9]. Until this century, the emerging masquelet technique [10] becomes a new Petunia for staging treatment due to its simple operation, few complications, low infection rate, rapid postoperative bone healing process, and early weight-bearing time [11, 12]. However, the one-stage treatment of chronic osteomyelitis has become a reality due to the increasing level of surgical debridement, the development of microsurgical techniques, together with the advent of novel antibiotic carriers and bone substitutes. In this study, 64 patients with localized osteomyelitis (cierny Mader type III) were selected for analysis after receiving tibial plateau fracture surgery in our hospital from July 2009 to January 2018. Each case is presented with observed clinical efficacy.

## Patients and methods

### Study design and inclusion and exclusion criteria

This study was conducted in Tongde Hospital of Zhejiang Province. We aimed to include patients who were diagnosed with type III osteomyelitis (according to the Cierny-Mader classification) following tibial plateau fracture surgery, with symptoms occurring for more than 10 weeks. This type of osteomyelitis presents attached or floating bone slices with obvious boundaries, which is often accompanied by the characteristics of type I and type II osteomyelitis. This can be extensively removed without leading to instability of the bone segments. Two experienced surgeons independently classified each patient according to the Cierny-Mader classification. Disputes were resolved by a third assessor. The establishment of a co-diagnosis was based on at least one of the following validation criteria [13]: supportive histology, microbiological culture on at least two suspicious sites, the presence of the similar pathogen, a definite sinus directly connected to the bone or to the implant, wound purulent drainage, or the presence of intraoperative pus. The exclusion criteria included patients with type I, type II, and type IV osteomyelitis of the tibial plateau, along with type III cases with histories of other injuries and knee joint disease with clinical symptoms before the injury. Patients with known hypersensitivity to caesium, vancomycin, or gentamicin were also excluded. In addition, patients who refused to accept the protocol and were not suitable for muscle flaps were excluded.

From July 2009 to January 2018, using information from electronic medical records, we selected 64 patients with osteomyelitis after tibial plateau fracture surgery. However, 29 of them were eventually excluded for the following reasons: 14 cases were type II, five cases were type IV, and ten cases were type III. Specifically, among those classified as type III, one case was complicated with osteofascial compartment syndrome in the right leg; one, pigmented villonodular synovitis of the knee joint; one, spinal cord injury; two, treated with free flaps; and five cases were directly sutured. The remaining 35 cases (25 males, 10 females), who accounted for 35 limbs, had a mean age at diagnosis of 54 (34–82) years. Their average follow-up time was 38 (24–60) months. In all the cases, the histopathological results revealed mature lamellar bone with focal degeneration, dense fibrous tissue hyperplasia, and inflammatory cell infiltration in the bone trabeculae. This finding was consistent with osteomyelitis. All treatment plans were approved by the ethics committee of the Zhejiang Academy of traditional Chinese medicine (No. KTSC093) and in accordance with the ethical standards laid down in the 1964 Declaration of Helsinki and its later amendments. Informed consent was obtained from all the patients.

Routine X-ray and CT scan films were taken before operation to evaluate the extent and degree of the osteomyelitis-induced bone defect. Blood examination, erythrocyte sedimentation rate, C-reactive protein level, and wound bacterial culture and drug sensitivity were also examined.

### Treatment

#### Preparation of vancomycin calcium sulfate sustained-release materials

The calcium sulfate used in all cases was Osteoset RBK (Resorbable Mini beam Kit; Wright Company). Five cc RBK was mixed with one g vancomycin and 160,000 u gentamicin to form 50 large particles (with a diameter of 4 mm) and 200 small particles (with a diameter of 2.8 mm). The dose of RBK was about 2 mL.

#### Operation proposal

All 35 cases underwent complete debridement prior to the procedure. Afterwhich, the size of the bone cavity and the area of the soft tissue defect were recorded. The scar tissues around the wound edge and the necrotic tissues were then excised. The sclerotic bones and free necrotic bones were also removed. As for the patients with severe bone sclerosis, the sclerotic bone was flattened with an orthopedic grinding drill until the bone surface and the soft tissue release the pus. The wound surface was then washed thrice with hydrogen peroxide and a large amount of normal saline. Finally, the beddings were also replaced. After the operation, the dead bone and necrotic soft tissue were sent for pathological examination and bacterial culture. Next, the autogenous iliac bone was granulated and then mixed with the vancomycin-loaded calcium sulfate. The particles were mixed evenly according to the optimized proportion before being implanted into the bone defect cavity.

#### Designing the gastrocnemius muscle flap

To create the lateral gastrocnemius muscle flap, a straight incision down to the subcutaneous tissue level was made on the posterolateral side of the leg. The lateral edge of the muscle flap was cut to locate the peroneal nerve. The space between the lateral head of the gastrocnemius and the soleus muscle was located at the posterior edge of the leg, which was bluntly separated. The incision was then extended up to the popliteal fossa. Following this, the origin of the lateral head of the gastrocnemius on the femoral condyle was dissected. The vascular pedicle of the lateral head of gastrocnemius was dissected to protect it. After that, the medial margin of the muscle flap was dissected in the internal and external space of the gastrocnemius muscle and the distal gastrocnemius was then severed.

As for the gastrocnemius medial head muscle flap, an incision was made in the medial leg to locate the space between the medial head of the gastrocnemius muscle and the soleus muscle. After the blunt separation of these muscles, the medial edge of the muscle flap was dissected along the medial space of the gastrocnemius muscle and was then amputated at the distal end of the gastrocnemius muscle. The kind of gastrocnemius muscle flap selected to cover the wound depended on the location of the wound. A free skin graft was specifically designed to cover the area of soft tissue defect in each patient.

#### Treatment after surgery

Patients were treated with anticoagulants, antibiotics, multimodal analgesia, and anticoagulation and antispasmodic therapy. All patients received intravenous antibiotics for 2 weeks and oral antibiotics for 4 weeks. The subsequent antibiotic selection was based on the culture and drug sensitivity results of the samples collected during the operation. If the culture result was negative, then the use of cephalosporin or clindamycin was continued. Rifampicin and/or quinolones were added to patients suspected of staphylococcal and/or Gram-negative bacteria-associated biofilm infections. Furthermore, their routine blood test, ESR, CRP, and other biochemical indices were re-examined on the 1st, 3rd, 7th, and 14th day. Bacterial culture of wound surface was done every 3 days. Antibiotic use was discontinued if the results have been negative in two consecutive tests. If the drainage volume was less than 5 mL and the bacterial culture in the drainage fluid was negative for two consecutive times, the drainage tube was pulled out and the time of removing the drainage tube was recorded. X-ray examination was performed regularly after operation, depending on the progress of the bone healing.

#### Clinical follow-up efficacy evaluation

All cases were followed up. The follow-up indicators included the drainage tube placement time, fracture healing time, infection control rate, and the incidence of bone nonunion and other complications. Hospital for Special Surgery knee score (HSS) was performed preoperatively and 2 years postoperatively, if possible.

#### Data processing and result analysis

Descriptive statistics were conducted using the Statistical Package for the Social Sciences (SPSS) 19.0 software (IBM, Armonk, NY, USA). The Kolmogorov-Smirnov test was initially used to evaluate the normal distribution of the continuous variables. The data are expressed as the mean (SD) and the median (range) of the normal distribution variables and non-normal distribution variables, respectively.

## Results

All patients were followed up for an average of 38 (24–60) months. No amputation was performed.

### Patient information

**Fig. 1:**
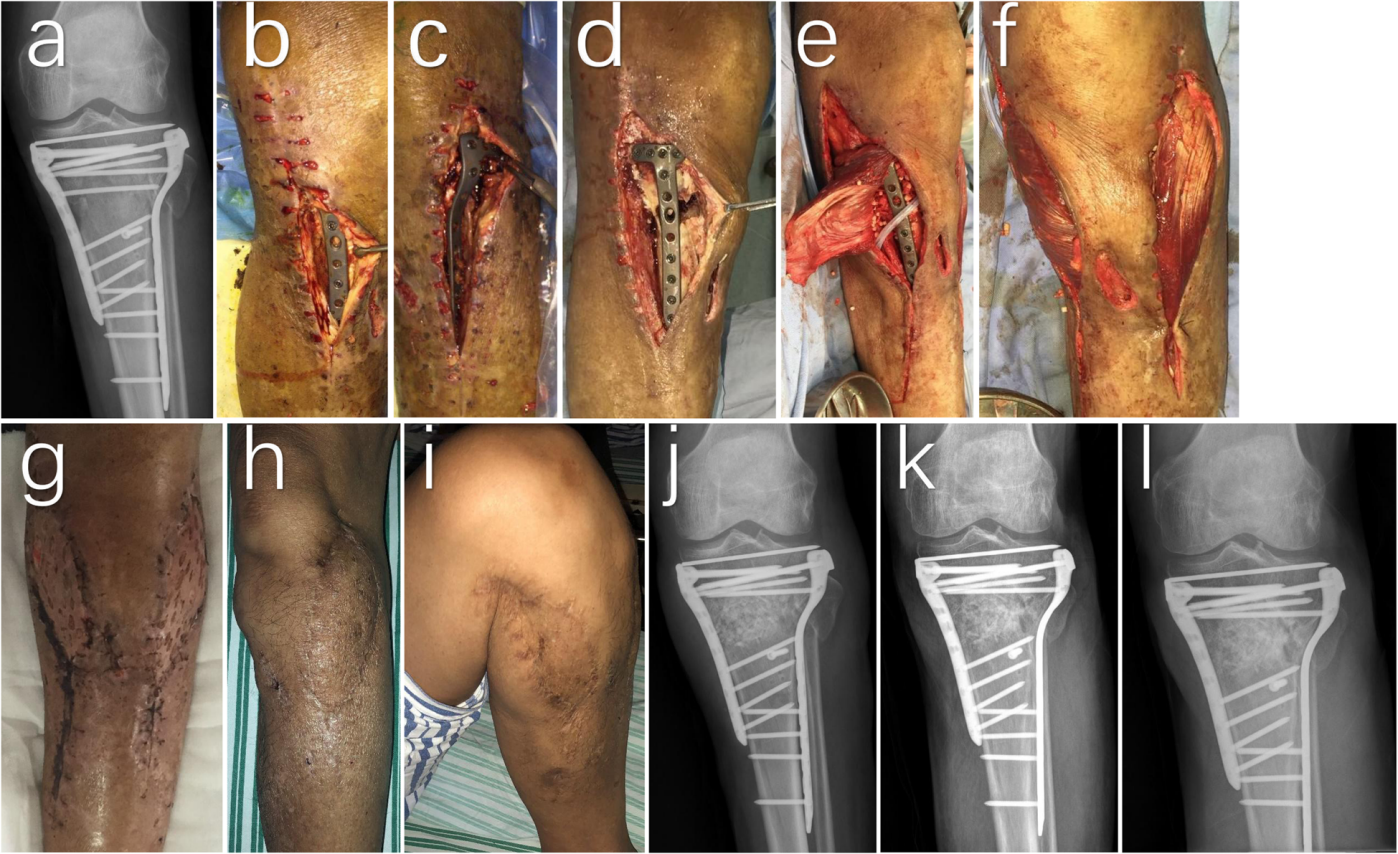
Typical case 1: Patient No. 14 presenting with plate exposure after tibial plateau fracture (Schatzker VI) surgery (**b**, **c**), The X-ray result shows the presence of local dead bone (**a**, red arrow). First, the necrotic tissue and inflammatory tissue (**b**, **c**) are completely removed. After removing the dead bone through the medial window, a 5 × 3 cm bone defect is found (**d**). The internal and external head muscle flaps of the gastrocnemius muscle are being designed and separated. The autologous iliac bone and the vancomycin/gentamicin-calcium sulfate-loaded artificial bone are being implanted into the bone defect (**e**). After covering the wound surface (**f**) of the leg with the internal and external cephalic muscle flaps from the gastrocnemius muscle, free skin grafting is performed on the surface of the muscle flap. Two weeks after operation, the skin graft survived and the incision healed well without swelling and exudation (**g**); The X-ray result shows that the bone graft was sufficient for the bone defect. The dead bone is also being removed (**j**). One year after operation, the patients recovered well (**k**). Two years after operation, the wound healed well and the knee joint flexion and extension function is good (**h**, **i**); The X-ray result shows that the fracture healed well (**l**)

Based on the Cierny-Mader classification, there were 20 cases of type A and 15 cases of type B. Among them, 18 cases were plate-exposed, 14 cases were due to sinus tracts, two were due to skin necrosis, and one was bone-exposed. Furthermore, there were18 and 17 cases of chronic osteomyelitis of the tibial plateau on the left and right leg, respectively. All cases were due to traumatic osteomyelitis after internal fixation due to tibial plateau fracture. Specifically, Schatzker type VI was the most common (21 cases), followed by Schatzker type V (five cases). As for the cause of fracture, the majority were due to traffic accidents (23 cases), followed by injury due to heavy objects (seven cases) and falling injury (five cases) (Table 1, Fig. 1).

**Table 1:** Clinical characteristics of the included patients

No.	Sex/age	C–M host classification	Side	Infection position	Local comorbidity	Schatzker type	Culture result
1	M/56	A	L	Lateral	Sinus tract	II	*Enterococcus faecalis*
2	M/54	A	L	Lateral	Sinus tract	II	*Staphylococcus aureus*
3	F/51	B	R	Medial	Plate exposed	VI	*Pseudomonas aeruginosa*
4	F/51	A	R	Lateral	Plate exposed	II	*Staphylococcus aureus*
5	M/81	B	L	Lateral	Skin necrosis	VI	
6	F/49	A	L	Medial	Sinus tract	IV	
7	F/82	B	L	Lateral	Plate exposed	II	
8	M/45	B	L	Lateral	Sinus tract	VI	
9	M/41	B	R	Medial	Plate exposed	VI	
10	F/64	A	L	Lateral	Plate exposed	V	*Staphylococcus epidermidis*
11	M/57	B	R	Medial	Plate exposed	VI	
12	M/63	A	R	Medial	Plate exposed	V	*Escherichia coli*
13	F/55	A	L	Lateral	Sinus tract	VI	
14	M/58	A	L	Medial Lateral	Plate exposed	VI	
15	M/34	B	R	Lateral	Plate exposed	V	
16	M/57	A	R	Lateral	Plate exposed	III	
17	M/71	B	L	Medial	Plate exposed	VI	
18	M/53	A	L	Lateral	Sinus tract	II	
19	M/54	A	L	Lateral	Plate exposed	VI	*Klebsiella pneumoniae*
20	M/54	B	L	Medial	Plate exposed	IV	
21	M/49	A	L	Medial Lateral	Sinus tract	VI	
22	F/56	B	R	Medial	Skin necrosis	VI	*Candida albicans*
23	M/64	B	R	Lateral	Sinus tract	VI	*Staphylococcus aureus*
24	M/54	A	L	Medial	Sinus tract	VI	
25	M/57	B	R	Medial	Bone exposed	V	*Streptococcus equisimilis*
26	M/45	A	R	Lateral	Plate exposed	VI	
27	F/44	A	L	Medial	Sinus tract	V	*Streptococcus equisimilis*
28	M/69	B	R	Medial	Plate exposed	VI	*Klebsiella pneumoniae*
29	F/49	A	R	Medial	Sinus tract	VI	
30	M/41	B	R	Medial	Sinus tract	VI	
31	M/44	A	L	Medial Lateral	Plate exposed	VI	
32	M/60	A	R	Medial	Plate exposed	VI	Methicillin-resistant *Staphylococcus aureus + Acinetobacter baumannii*
33	F/35	A	R	Lateral	Sinus tract	II	
34	M/44	A	R	Medial	Sinus tract	VI	*Staphylococcus aureus*
35	M/47	B	L	Medial	Plate exposed	VI

### Inflammatory biomarkers and microbiology

The cases’ average serological levels of WBC, ESR, and CRP were 7.5 (normal value: 4.4–13) × 10^9^/L, 17 (4–110) mm/h, and 5.1 (0.2–198) mg/L, respectively. Abnormal serological levels on the previously mentioned biomarkers were recorded in 9/35, 17/35, and 14/35 patients, respectively. Among the 35 cases, 14 were positive for pathogen culture (13 cases of which were due to a single bacterial etiologic agent and one case was due to a multibacterial infection), while one was positive for fungal infection. *Staphylococcus aureus* (four cases) was the most common pathogen, followed by *Klebsiella pneumoniae* (two cases) and *Streptococcus equisimilis* (two cases) (Table 1).

### Management of internal fixation

In this study, the internal fixation was dismantled and changed to the external fixation stent due to severe infection among four patients (No. 2, 6, 24, 32). Seven patients (No. 17, 23, 25, 27, 29, 30, 33) had their internal fixation dismantled in other hospitals after the fracture had healed. Due to severe infection and healed fracture partly, the internal fixation was dismantled to change the brace among four patients (No. 4, 5, 12, 13), and the internal fixation was preserved with the remaining patients. Retention and removal criteria are listed in Table 2.

**Table 2:** Retention and removal criteria

Retention criteria	
●Remove the infected lesion completely	
●Internal fixation does not fail after debridement	
●The tight fit between the plate and the bone surface or the presence of voids can be eliminated	
●Soft tissue coverage with good vascularization	
●Autologous iliac bone is able to adequately fill the dead space	
●Local application of sustained drug release	
●A sufficient course of antibiotics should be administered systemically	
Removal criteria	
●Internal fixation fails after debridement	
●Existence of large void between the plate and the bone surface	
●Traumatic osteomyelitis with a course of more than 2 months with partial or complete healing of the fracture	
●Extensive and severe infection	
●Intramedullary fixation	

### Repair of soft tissue defect

As for the flaps used, 17 cases were covered with the medial head of the gastrocnemius alone, 15 cases with the lateral head of gastrocnemius alone, and three cases were covered with a combination of the medial and lateral heads of the gastrocnemius. All patients were covered with free skin grafts. Based on the follow-up, all the muscle flaps and free skin grafts inserted survived. The average time of drainpipe laying was 14 (10–20) days, while the average bone healing time was 27 (16–60) weeks (Tables 3 and 4).

**Table 3:** Clinical efficacy of the intervention on included patients

No.	Sex/age	Follow-up (M)	The time of laying drainpipe(D)	Bone healing time (W)	Complications	HSS score		
						Before surgery	1 year after surgery	2 years after surgery
1	M/56	26	11	18		66	89	90
2	M/54	28	12	16		68	91	91
3	F/51	48	14	24	Iliac hematocele	53	87	88
4	F/51	32	13	19		63	90	90
5	M/81	44	15	32		50	85	87
6	F/49	42	12	24		54	81	85
7	F/82	30	19	22		65	89	90
8	M/45	56	10	28	Aseptic wound leakage	52	83	85
9	M/41	58	14	21		49	87	87
10	F/64	42	11	20		56	85	86
11	M/57	34	13	28	Iliac anterolateral numbness	51	82	86
12	M/63	58	15	60	Nonunion	44	81	85
13	F/55	37	10	25		48	87	87
14	M/58	43	18	36	Iliac anterolateral numbness	35	80	85
15	M/34	41	12	23		48	89	89
16	M/57	36	10	19		61	88	89
17	M/71	49	15	29		50	85	86
18	M/53	24	10	21		59	90	90
19	M/54	46	13	23	Iliac hematocele	62	87	88
20	M/54	45	14	25		56	90	90
21	M/49	42	16	28	Iliac anterolateral numbness	38	78	83
22	F/56	30	18	33		46	81	85
23	M/64	28	17	26		45	87	88
24	M/54	24	15	31		53	86	89
25	M/57	47	14	24		49	88	89
26	M/45	29	15	38	Iliac anterolateral numbness	46	87	90
27	F/44	26	12	23		54	89	89
28	M/69	32	13	26		58	83	85
29	F/49	33	14	30		59	82	84
30	M/41	25	13	25		49	86	86
31	M/44	28	20	32	Iliac anterolateral numbness	43	79	85
32	M/60	60	16	26	Infection relapse	57	72	78
33	F/35	48	10	20	Infection relapse	69	74	80
34	M/44	35	14	27		60	86	89
35	M/47	31	15	41		62	87	88

*HSS* Hospital for Special Surgery knee score

**Table 4:** Result after followed up

Follow up time, mouths	38 (24~60)
Complications, *n* (%)	
Relapse	2 (5.71)
Nonunion	1 (2.86)
Aseptic exudate	1 (2.86)
Anterolateral numbness of the iliac thigh	5 (14.29)
Hematocele in the iliac bone area	2 (5.71)
Total	11 (31.43)
Average time of drainpipe laying, days	14 (10–20)
Average bone healing time, weeks	27 (16–60)
Eradication rate of osteomyelitis, %	94.29
Hospital for special surgery knee score	
One-year follow-up	86 (72–91)
Two-year follow-up	87 (78–91)

### Infection eradication rate and adverse events

The average implantation volume of the autogenous iliac bone mixed with the calcium sulfate artificial bone was 21 (7–42) mL. All patients were covered with free skin grafts. All the inserted muscle flaps and free skin grafts survived. The wounds were covered using the medial head of the gastrocnemius muscle in 17 cases, the lateral head in 15 cases, and the combination of the two in three cases. The eradication rate of osteomyelitis was 94.29% (33/35). Two cases experienced relapse, of which 1 case had repeated exudation within 2 years and the other case, within 3 years after operation. Both of them were cured after re-expansion and implantation of the vancomycin/gentamicin-calcium sulfate and autogenous iliac bone. Nonunion occurred in one case, but the infection was controlled and healed after bone grafting. One case of aseptic exudate was cured after changing the dressing. Anterolateral numbness of the iliac thigh occurred in five cases, but these were relieved after 3 to 6 months. Lastly, two cases of hematocele in the iliac bone area were cured after extrusion and dressing change.

### HSS (Hospital for Special Surgery knee score)

The average HSS (Hospital for Special Surgery knee score) of the 35 patients was 54 (35–69) points before operation, which improved to 86 (72–91) points at the 1-year follow-up and 87 (78–91) points at the 2-year follow-up (Tables 3 and 4).

## Discussion

Chronic osteomyelitis can occur after internal fixation of plates to fix tibial plateau fracture. Among our cohort, especially those classified with a Schatzker VI tibial plateau fracture (21/35), poor wound healing was the most common complication. In general, the treatment can be very challenging, and when it fails, its consequences are quite serious. This is because a poorly-healed wound that becomes infected may lead to complications, such as plate exposure, infective osteomyelitis, or nonunion of the bones [14]. It is difficult to completely cure this type of wound with just irrigation, drainage, wound expansion, and dressing, as the wound might expand in size. As such, special attention must be provided to avoid this.

At present, the clinical treatment of osteomyelitis with a bone defect is mainly divided into staged treatment and one-stage treatment. Masquelet technique is a common method for a staging treatment. In a systematic review of 48 studies, 1386 patients were treated with induced membrane technology, and the infection control rate was about 90%. The average time of bone healing was 6.6 months, and the incidence of nonunion was 13% [15]. Comparing to a staged treatment, one-stage treatment’s primary treatment period was shorter, the number of operation was less, the infection control rate and bone healing rate were no less than the former, and the economic cost was lower. In a survey of 505 patients with chronic osteomyelitis, the total effective rate of one-stage treatment with muscle flap, bioceramic (including calcium sulfate), or bioactive glass in dead space of all patients reached 87.5–94.2% [16]. In another recent report, the total effective rate of one-stage treatment of osteomyelitis with the same method was close to almost 95% [17]. The infection eradication rate of our study also reached 94.29% (33/35 patients), while the incidence of nonunion was 2.86% (1/35 patients) and the average bone healing time was 27 weeks (16–60 weeks). In a retrospective study of Jiang et al.’s one-stage treatment for localized calcaneal osteomyelitis with calcium sulfate alone, the infection eradication rate was 85.3% (29/34 patients). But the incidence of aseptic wound exudation was 32.35% (11/34 patients) [18]. Comparatively, our incidence rate of aseptic wound exudation was 2.86% (1/35 patients), which was lower than previous studies whose incidence rates ranged from 6.00% (6/100) [19] to 33.33% (7/21) [20]. We believe that the low incidence rate of aseptic wound exudation among our patients may be related to our fewer implantation of the antibiotic-calcium sulfate. In addition, the primary treatment with calcium sulfate hydroxyapatite biological composite as dead space filling material has achieved an infection control rate close to ours, reaching 92.3–96%. But the incidence has about 11% nonunio n[19, 21, 22]. In addition, some scholars claimed that for the bone defect after debridement of chronic osteomyelitis, one-stage allogeneic bone transplantation was performed, and the resulting effective rate was 91.43%. They believe that chronic osteomyelitis should not be seen as the contraindication of one-stage allogeneic bone transplantation [23]. However, this is still under discussion with a lack of supporting evidence, and there were some problems in this study where 8.6% (3/35) patients had severe rejection.

The development of one-stage treatment benefits from the antibiotic calcium absorbable drug delivery system (typically calcium sulfate), which has been used in the clinical treatment of bone infectious diseases with satisfying clinical results [22, 24, 25]. Many kinds of antibiotics can be combined with calcium sulfate. After implantation, the antibiotics would slowly be released into the lesion as the calcium sulfate becomes hydrolyzed. Locally, these antibiotics then consolidate and maintain high concentrations to inhibit bacterial growth to control infection. These anti-bacterial effects can last for about two months [26–29]. Other than the antibiotic activity, calcium sulfate is implanted into the bone defect cavity to promote the proliferation of local microvessels and increase the expression of various growth factors. The release of calcium during hydrolysis accelerates the formation of local new bones by promoting the proliferation of osteoblasts and inhibiting the activity of osteoclasts. This effect is very beneficial in hastening the healing process of bone defects [30, 31]. Currently, many studies have focused on improving the bone induction characteristics of the implanted artificial bones in order to improve the bone healing rate [27, 32, 33]. Ferguson reports that a gentamicin-eluting synthetic bone graft substitute is effective in managing dead-space in surgically treated chronic osteomyelitis with a 4.3% infection recurrence rate and a 73.8% bone void-filling rate [34]. It has been reported that using such a system can maintain a high local concentration of antibiotics. However, an excessive concentration of antibiotics can inhibit osteoblasts, which may also be one of the causes for bone nonunion [35]. We mainly implanted the autogenous iliac bone into the bone defect cavity, and calcium sulfate was only used as a sustained release drug carrier to reduce the total amount of implantation. This had the benefit of maintaining the optimal concentration of local antibiotics to prevent these from inhibiting the activity of osteoblasts.

There have been a few reports on the experimental and clinical application of antibiotic-loaded calcium sulfate combined with autologous bone implantation. Autogenous cancellous bone graft has always been the “gold standard” for bone defect treatments [36] due to its superiority to any other bone substitutes in improving the bone healing rate. The cancellous graft is usually derived from the iliac bone. The cancellous bone graft has the advantage of rapid revascularization and thorough integration with the bone structure. However, for the purpose of infection treatment, the implantation of autologous bone after debridement remains controversial.

The limitation of this study lied in the lack of sample size and the lack of strict randomized controlled trials. Due to the small sample size, the statistical ability of this study was relatively weak. It can be further improved by conducting a multicentre randomized controlled double-blind trial.

To conclude, we were able to achieve satisfactory results in treating localized osteomyelitis after tibial plateau fracture by using thoroughly removing the focus of infection, implanting an antibiotic-calcium sulfate artificial bone combined with autologous iliac bone graft, and by placing a gastrocnemius muscle flap with a rich blood supply and strong anti-infection to fill the wound cavity to repair the defect and close the wound.

## Data Availability

The datasets used or analyzed during the current study are available from the corresponding author on reasonable request.

## Abbreviations

HSS: Hospital for Special Surgery knee scores
RBK: Resorbable Mini beam Kit
SPSS: Statistical Package for the Social Sciences
SD: Standard deviation
WBC: White blood cell
ESR: Erythrocyte sedimentation rate
CRP: C-reactive protein
